# Flexible Transparent Electrode Characteristics of Graphene Oxide/Cysteamine/AgNP/AgNW Structure

**DOI:** 10.3390/nano10122352

**Published:** 2020-11-27

**Authors:** Junhwan Jang, Ju-Young Choi, Jihyun Jeon, Jeongjun Lee, Jaehyuk Im, Jaegun Lee, Seung-Won Jin, Hyeong-Joo Park, Seung-Hyun Lee, Dam-Bi Kim, Chan-Moon Chung, Soohaeng Cho

**Affiliations:** 1 Department of Physics, Yonsei University, Wonju, Gangwon-do 26493, Korea; jgh6758@yonsei.ac.kr (J.J.); jihyunn1211@yonsei.ac.kr (J.J.); leejj0858@yonsei.ac.kr (J.L.); jaehyuk5457@yonsei.ac.kr (J.I.); jay0gun@yonsei.ac.kr (J.L.); 2 Department of Chemistry, Yonsei University, Wonju, Gangwon-do 26493, Korea; cjy0510@yonsei.ac.kr (J.-Y.C.); jinsw0906@yonsei.ac.kr (S.-W.J.); hyeongjoo1016@yonsei.ac.kr (H.-J.P.); sh_lee2495@yonsei.ac.kr (S.-H.L.); dam818@yonsei.ac.kr (D.-B.K.)

**Keywords:** silver nanowire, GO-cysteamine-AgNP (GCA), transparent and flexible electrode, resistive switching memory

## Abstract

Graphene oxide (GO)–cysteamine–Ag nanoparticles (GCA)–silver nanowire (AgNW) fabricated by depositing GCA over sprayed AgNWs on PET films were proposed for transparent and flexible electrodes, and their optical, electrical, and mechanical properties were analyzed by energy-dispersive X-ray spectroscopy, Fourier-transform infrared spectroscopy, Raman spectroscopy, atomic force microscopy, scanning electron microscopy, transmission electron microscopy, current-voltage measurements, and bending test. GCA–AgNW electrodes show optical transmittance of >80% at 550 nm and exhibit a high figure-of-merit value of up to 116.13 in the samples with sheet resistances of 20–40 Ω/◻. It was observed that the detrimental oxidation of bare AgNWs over time was considerably decreased, and the mechanical robustness was improved. To apply the layer as an actual electrode in working devices, a Pt/GO/poly(3,4-ethylenedioxythiophene) polystyrene sulfonate/GCA–AgNW/polyethylene terephthalate structure was fabricated, and resistive switching memory was demonstrated. On the basis of these results, we confirm that the proposed GCA–AgNW layer can be used as transparent and flexible electrode.

## 1. Introduction

There is an increasing demand for transparent conductive oxide (TCO) films for applications such as liquid crystal displays, plasma display panel, organic electro luminescence displays (OLEDs), solar cells, and electromagnetic wave shielding films. Indium tin oxide (ITO) has been typically used as TCO film [1] because of its low electrical resistivity and high transmittance [2]. However, ITO has problems (e.g., high price of raw material indium, high manufacturing cost, and characteristic change owing to deterioration when exposed to plasma during processing) that need to be overcome [3,4]. Carbon nanotubes and nanowires (NWs), as alternative materials, have attracted considerable attention [5,6,7,8]. In addition, the demand for lightweight, compact displays and touch panels to supplement the fragile nature of glass is increasing. Polymer substrates are being actively researched as a new substrate, and the necessity for research to apply transparent conductive electrodes to polymer substrate is greatly increased. The polymer material substrate includes polyethylene–terephthalate (PET) and polyethylene naphthalate (PEN), which have been proposed for use as TCO films [9,10,11].

In this study, we worked on the next-generation transparent electrode using silver nanowires (AgNWs). AgNWs mesh layer as a transparent electrode exhibited high optical transparency and was flexible [12,13,14]. In addition, AgNW has the advantage of mass production through solution processes [15,16]. There are various methods of forming a AgNW mesh film such as spray coating [17], spin coating, dip coating [18], slit coating [19], and bar coating [20]. In this study, AgNW electrodes were fabricated using a spray coater. The spray process has the advantage of uniform deposition compared to bar coating or dip coating because it sprays the solution from above without contacting the substrate. Light transmittance and surface roughness of the AgNW mesh film depend on the wire density. An increase in wire density to achieve high electrical conductivity lowers the transmittance and increases the surface roughness [15]. This increase in surface roughness affects leakage current generation and interface resistance when manufacturing thin film electrical devices (e.g., displays) and adversely affects the substrate adhesion [12]. In addition, AgNW layer has reduced electrical conductivity because the wire mesh structure is broken owing to oxidation or corrosion problems of AgNW surface due to oxygen in the atmosphere [21].

To overcome these limitations, in this study, GO–cysteamine–AgNPs (GCA) structure was synthesized and coated on AgNW films to improve the resistance to surface oxidation and mechanical degradation. GCA–AgNW layers were fabricated with various ratios of cysteamine group and Ag nanoparticles (AgNPs), and enhanced electrical and mechanical properties were comprehensively characterized. In addition, to confirm the applicability of the fabricated GCA–AgNW electrode to flexible memory, a resistive switching memory (ReRAM) with the Pt/GO/ poly(3,4-ethylenedioxythiophene) polystyrene sulfonate (PEDOT:PSS)/GCA–AgNW structure was fabricated, and the memory operation was demonstrated.

## 2. Materials and Methods

### 2.1. Material Preparation

**Scheme 1:**
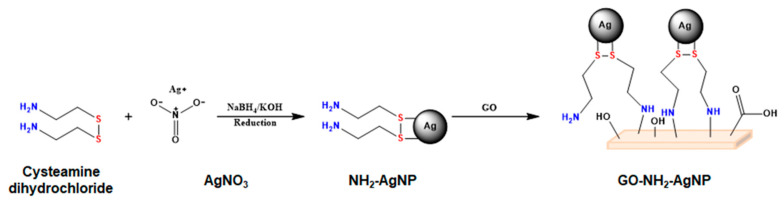
Synthesis process of Graphene oxide(GO)–cysteamine–Ag nanoparticles (GCA).

AgNW solution (DT–AgNW–N30–1EOH, ditto technology Co. Korea) of 1% by weight in ethanol was diluted to obtain the ratio of ethanol:AgNW = 9:1 wt.%. GCA was synthesized as follows [22]. First, AgNO_3_ (0.0100 g, 6 × 10^−5^ mol) was stirred in distilled water (5 mL) at room temperature for 5 min, followed by the addition of cysteamine dihydrochloride (0.0135 g in 2 mL of distilled water). After stirring for 15 min at 10 °C, the cooled solution of 0.1 mol L^−1^ NaBH_4_ in 20 mmol L^−1^ KOH (0.1 mL) was added dropwise to the resulting mixture. After stirring for 30 min at 10 °C, the resultant mixture was left for 24 h without stirring to form a dark reddish-brown nanoparticle suspension. The ratios of AgNO_3_ and cysteamine dihydrochloride in Cys–AgNPs were 1:1, 1.25:1, 1.5:1, or 2:1. GO (0.05 g) was homogeneously dispersed in 25 mL of distilled water by tip sonication for 30 min. The obtained Cys–AgNPs suspension was added to the dispersed GO suspension, and the mixture was refluxed and stirred for 24 h. Then, the resulting product was centrifuged, washed with distilled water and ethanol many times, and dried in a vacuum oven at 50 °C overnight. Finally, GO-based composites covalently linked with Cys–AgNPs (GCA) powder were first dispersed in 25 mL of distilled water by tip sonication for 30 min. The GCA/H2O suspension was diluted with 25 mL of isopropyl alcohol (IPA) by an additional tip sonication for 30 min to yield the final GCA suspension (Scheme 1). The finished GCA solution was expressed as GCA_1:1_, GCA_1.25:1_, GCA_1.5:1_, and GCA_2:1_ according to the ratio of cysteamine and AgNP, respectively. PEDOT:PSS (high conductivity grade, #655201, Sigma-Aldrich, St. Louis, MO, USA) and GO used in memory fabrication were used without additional purification.

### 2.2. Analytical Methods

AgNWs were deposited using a spray coater (TAD-400SR, BV-500, Banseok Co., Seoul, Korea). GCA coating was performed using a spin-coater (ACE-200, Dong Ah Trade Co., Seoul, Korea). Scanning electron microscopy (SEM, a Quanta 250 FEG electron microscope from FEI Co., Hillsboro, OR, USA) was used to analyze the surface structure of AgNWs and GCA–AgNW films. The SEM measurement conditions were 30,000 magnification at the acceleration voltage of 20 keV and spot size of 3.5. In order to measure the surface roughness of the thin film, atomic force microscopy (AFM, NX10 atomic microscope from Park system Co., Suwon, Gyeonggi-do, Korea) was employed. Cross-sectional images of GCA–AgNW were obtained using transmission electron microscopy (TEM, JEM-F200, JEOL Ltd. Co., Akishima, Tokyo, Japan), and the components of GCA and GO were compared using energy dispersive X-ray spectrometry (EDS, Berwyn, PA, USA). To obtain the transparency of the electrode, transmittance in the visible light region was measured by UV-Vis spectroscopy (Lambda 25, Perkin Elmer Co., Waltham, MA, USA). The light source was a tungsten–halogen lamp with the wavelength range of 300–800 nm. The electrical properties of electrode elements were obtained by attaching silver paste to the center of the four sides. Surface resistance was determined using a Hall-effect measurement system (HMS-3000, Ecopia Co., Anyang, Gyeonggi-do, Korea). Current–voltage (I–V) characteristics were evaluated using a Keithley 2634 source meter (Keithley Inc., Cleveland, OH, USA) under ambient air and temperature conditions.

### 2.3. GCA-AgNW Electrode Fabrication Process

**Scheme 2:**
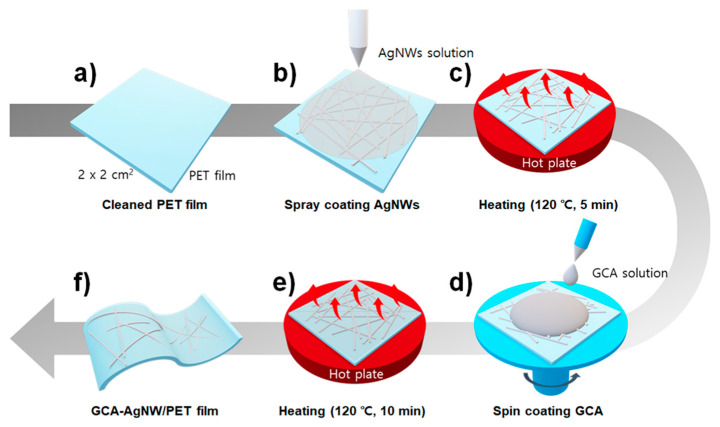
GCA-AgNW electrode fabrication process: (**a**) PET film cleaning, (**b**) AgNWs spray coating, (**c**) heating at 120 ℃ during 5 min, (**d**) GCA spin coating, (**e**) heating at 120 ℃ during 10 min, (**f**) GCA-AgNW/PET film.

Scheme 2 shows the schematics of the manufacturing process of GCA–AgNW electrodes. The PET substrate was cut into 2 × 2 cm^2^ pieces and cleaned. The cleaning process involved sequential ultrasonic cleaning for 3 min in acetone, IPA, and DI-water followed by N_2_ gas blowing. The AgNW (20–40 nm diameter, 10–20 μm length) solution was sprayed for 1 s on the cleaned PET with the N_2_ gas pressure of 1.5 bar, air pressure of 3 bar, 15 cm spacing between the substrate and the spray valve, and a valve scale of 1 (Scheme 2b). The samples were heated on a hot plate for 5 min at 80 °C to evaporate the solvent in the AgNW solution (Scheme 2c). Scheme 2d shows the deposition of GCA solution onto AgNW/PET using a spin coater. A total of 10 μL of the GCA solution was spin-coated for 10 s at 300 rpm, 10 s at 1000 rpm, and 40 s at 2000 rpm. To achieve stable bonding of GCA and AgNW via the Scheme 2e process, heating was performed at 120 °C for 10 min.

### 2.4. Pt/GO/PEDOT:PSS/GCA-AgNW/PET Memory Fabrication Process

**Scheme 3:**
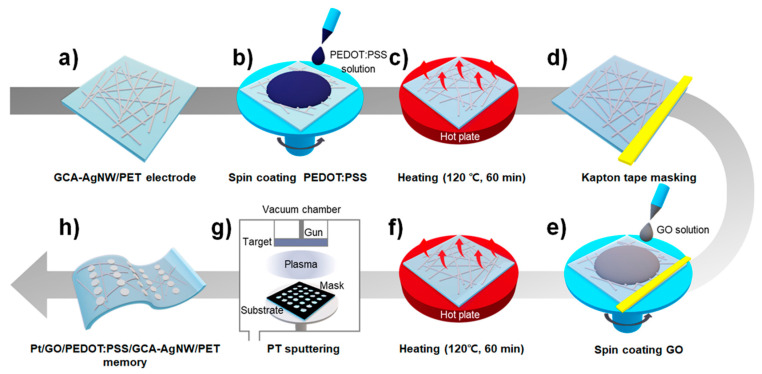
Schematics of the fabrication process of the Pt/GO/PEDOT:PSS/GCA–AgNW/PET memory structure: (**a**) GCA-AgNW/PET electrode, (**b**) PEDOT:PSS spray coating, (**c**) heating at 120 ℃ during 60 min, (**d**) Kapton tape masking (**e**) GO spin coating, (**f**) heating at 120 ℃ during 60 min, (**g**) Pt sputtering, (**h**) Pt/GO/PEDOT:PSS/GCA-AgNW/PET memory.

To demonstrate the functional application of the GCA–AgNW electrode for use in flexible nonvolatile resistive switching memory, a memory device with Pt/GO/PEDOT:PSS/GCA–AgNW/PET structures was fabricated. GO was used as the resistive change layer [23]. The PEDOT:PSS layer was deposited by spin coating to improve the surface uniformity of the lower electrode [24]. PEDOT:PSS and GO layers were deposited by spin coating for 60 s at 2000 rpm and 1500 rpm (Scheme 3b,e), respectively, and were heated at 80 °C for 60 min (Scheme 3c,f). The PET substrate may be damaged during the thermal evaporator process. Lastly, a metal shadow mask with holes having a diameter of 1 mm was attached on the GO thin film to form circular top Pt electrodes; then Pt was deposited at the pressure of 10^−3^ Torr using DC sputtering.

## 3. Results and Discussion

**Figure 1:**
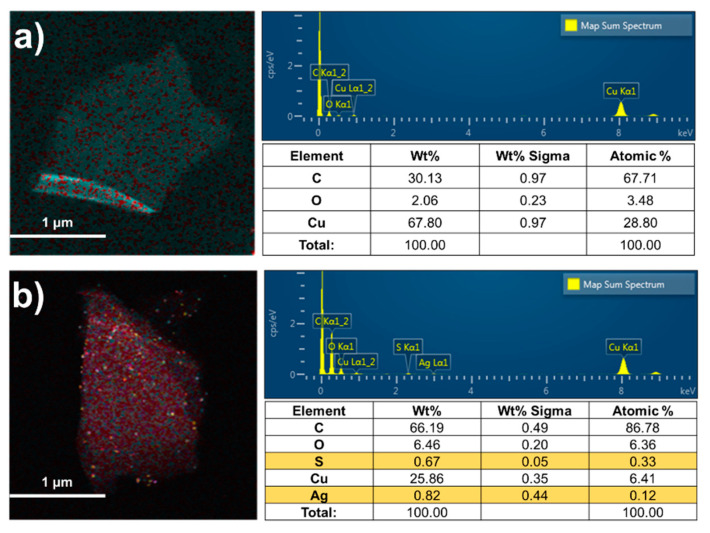
EDS analysis. (**a**) GO, (**b**) GCA_1:1_-AgNW.

Figure 1a,b show the EDS analysis data for GO and GCA_1:1_–AgNW, respectively. S and Ag, which are not observed in GO, are detected in GCA_1:1_–AgNW, indicating that cysteamine and AgNP are attached well to GO. The Raman spectra of GO and GCA are shown in Figure S1. GO exhibited two characteristic peaks (i.e., D and G bands at 1346 cm^−1^ and 1605 cm^−1^), which can be attributed to the symmetric A_1g_ vibration mode and E_2g_ vibration mode of sp^2^ carbon atoms, respectively. GCA showed two peaks, i.e., D and G bands at 1354 cm^−1^ and 1605 cm^−1^, respectively. The intensity ratio (I_D_/I_G_) of the D-band (I_D_) and G-band (I_G_) is commonly used to evaluate defects and disorder in the graphite structure, which is related to the degree of functionalization. The I_D_/I_G_ ratio of GCA slightly increased from 1.10 to 1.14 compared to that of GO. This result indicates that the ordered structure of GO is disrupted by the introduction of cysteamine–AgNPs. The chemical structures of GO and synthesized GCA were confirmed by Fourier-transform infrared spectroscopy (FT-IR), and the spectra are shown in Figure S2. The FT-IR spectrum of GO showed several characteristic bands at 3421, 1720, 1617, 1221, and 1047 cm^−1^, which correspond to the O–H group, carboxyl C=O, C=C skeleton, hydroxy C–OH and epoxy C–O–C stretching vibrations, respectively. However, in the spectrum of GCA, the intensity of the epoxy C–O–C stretching of GCA at 1047 cm^−1^ considerably decreased compared to that of GO. This result clearly confirmed that cysteamine was attached to the GO surface through nucleophilic attack by amine of cysteamine on epoxy groups.

**Figure 2:**
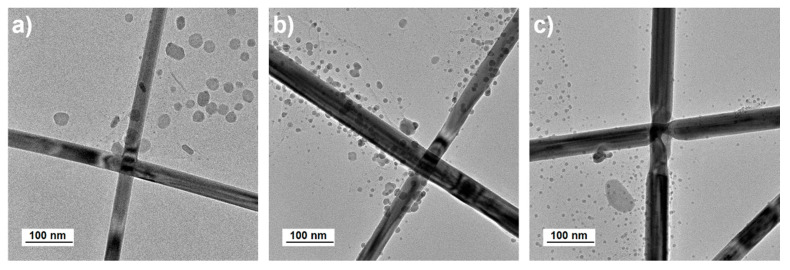
GCA-AgNW TEM image. (**a**) GCA_1:1_-AgNW not annealed, (**b**) annealed GCA_1:1_-AgNW, (**c**) annealed GCA_2:1_-AgNW.

Figure 2 shows the TEM images of GCA–AgNW layers. Figure 2a shows the TEM image of GCA_1:1_–AgNW that was not annealed. The images of annealed GCA_1:1_–AgNW and GCA_2:1_–AgNW are shown in Figure 2b,c respectively. The annealing process was performed for 5 min at 120 °C. The abovementioned images show that, using heat treatment, the AgNW–AgNW junction and AgNP–AgNW in the GCA solution become stronger. GCA_2:1_–AgNW has a smaller AgNP size than GCA_1:1_–AgNW. This result indicates that the size of AgNP is controlled according to the ratio of cysteamine in the GCA solution, which confirms that the ratio of cysteamine is one of the causes of change in performance.

**Figure 3:**
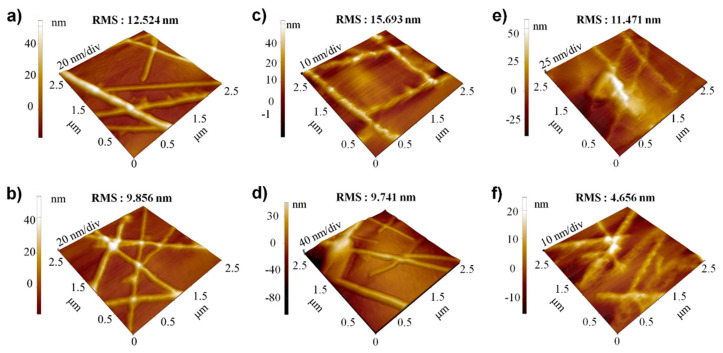
AFM images. (**a**) AgNWs before annealing, (**b**) annealed AgNWs, (**c**) GCA_1:1_–AgNW before annealing, (**d**) GCA_1:1_–AgNW after annealing, (**e**) GCA_2:1_–AgNW before annealing, (**f**) GCA_2:1_–AgNW after annealing.

Surface morphology was investigated by AFM, and Figure 3 shows images and the corresponding root mean square roughness (RMS) values for (a) AgNWs before annealing, (b) annealed AgNWs, (c) GCA_1:1_–AgNW before annealing, (d) annealed GCA_1:1_–AgNW, (e) GCA_2:1_–AgNW before annealing, and (f) annealed GCA_2:1_–AgNW. RMS was measured in the area of 2.5 × 2.5 μm^2^ and calculated by averaging the results of five or more measurements of the sample. Surface morphology explains that when AgNWs are covered with GCA, the RMS value is slightly increased. However, the annealing process clearly reduced the RMS roughness value.

**Figure 4:**
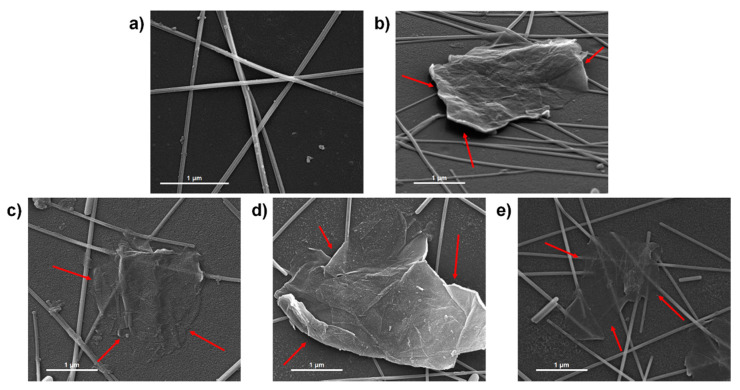
SEM images. (**a**) AgNWs on PET, (**b**) GCA_1:1_–AgNW before annealing, (**c**) annealed GCA_1:1_–AgNW, (**d**) GCA_2:1_–AgNW before annealing, (**e**) annealed GCA_2:1_–AgNW.

We also investigated the surface morphology by SEM, as shown in Figure 4. It is clear that GCA is deposited on the AgNW junction. Figure 4b and d shows that GCA is not properly attached to the substrate and AgNWs before the heat treatment. However, the annealing process strengthens the bonding between AgNW–AgNW junctions and especially between AgNW and substrate, as shown in Figure 4c and e, which indicates that the annealed GCA acted like an adhesive film covering AgNW/PET layers. It is in good agreement with the reduced average AFM RMS after annealing, as shown in Figure 3.

**Figure 5:**
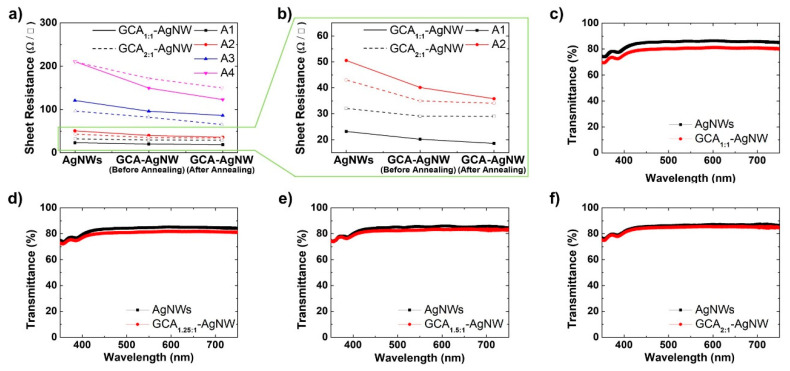
Sheet resistance and transmittance of AgNWs and GCA–AgNW. (**a**) Sheet resistance of all samples and the (**b**) sheet resistance of A1 and A2 group samples; (**c**–**f**) optical transmittance of AgNWs and GCA_1:1_–AgNW, AgNWs and GCA_1.25:1_–AgNW, AgNWs and GCA_1.5:1_–AgNW, and AgNWs and GCA_2:1_–AgNW, respectively.

Figure 5 shows the measured data of sheet resistances and UV-Vis to confirm the improvement in sheet resistance and transmittance when GCA–AgNW was produced by depositing GCA on the AgNW film. The various sheet resistances were achieved by changing the number of repeated spray injections (3–15 times). Figure 5b shows the comparison of changes in sheet resistances of A1 and A2 samples. Apparently, when GCA is deposited on the AgNW film, the sheet resistance decreases, and it decreases more at high sheet resistance. After annealing, the sheet resistance generally decreases further, which results from the strengthened AgNW-AgNW and AgNW-substrate bonding as described previously with Figure 4c,e. Figure 5c is the transmittance change when GCA_1:1_ is deposited, and Figure 5d–f summarizes the change in transmittance of AgNWs covered by GCA_1.25:1_, GCA_1.5:1_, and GCA_2:1_, respectively. Although the transmittance in general was slightly lower owing to GO and AgNP contained in GCA, all GCA–AgNW samples exhibited transmittance values higher than 80% at 550 nm.

(1) $$\frac{Z_{0}}{2R_{s}}\frac{\sqrt{T}}{1-\sqrt{T}}$$

We calculated figures of merit (FOMs), as expressed by Equation (1) to evaluate the performance of transparent electrodes [25,26]. *Z*_0_ is the impedance in free space (337 Ω), *R_s_* is the sheet resistance, and T is the optical transmittance at 550 nm. For GCA_1:1_, FOM is increased by approximately 8.92% from 64.60 to 70.36, while the increment is 1.69% from 79.09 to 80.43 for GCA_1.25:1_. In addition, FOM for GCA_1.5:1_ is improved by approximately 7% from 108.54 to 116.13 and by 3.75% from 93.48 to 96.98 for GCA_2:1_. It was interpreted that the FOM value is generally improved owing to a decrease in sheet resistance rather than a decrease in transmittance. It was reported that a FOM value of 35 of more is sufficiently high enough for industrial application [27]. These results confirm all the GCA–AgNW samples with high FOM values can be used transparent electrodes.

**Figure 6:**
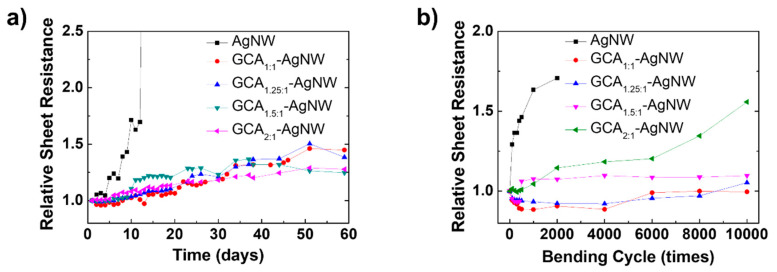
Mechanical characteristics of AgNWs and GCA–AgNW. (**a**) Sheet resistance stability test for AgNWs and GCA–AgNW. (**b**) Bending test results for AgNWs and GCA–AgNW.

To test sample robustness, we recorded the time dependence of sheet resistance for 60 days at room temperature and atmospheric pressure, as shown in Figure 6a. Three to five samples for each type were prepared to measure the average sheet resistance, and the rates of increase in sheet resistance are shown with the initial sheet resistance set to 1. For bare AgNWs, sheet resistance increased abruptly by ~400% only after 13 days, which was due to oxidation. However, all GCA–AgNW samples showed a less than 35% increase in sheet resistance even after 60 days. This clearly shows that the deteriorating oxidation process can be effectively suppressed by GCA. In addition, it is expected that GO and AgNP particles in GCA assist AgNW–AgNW binding, which increases chemical resistance to oxidation.

Figure 6b shows the mechanical bending test results of AgNWs and GCA–AgNW device bending tests. The bending radius was 4 mm, and the test was repeated 10^4^ times. For AgNW, sheet resistance could not be measured after 2 × 10^3^ cycles presumably owing to the collapse of the AgNW network. However, for GCA–AgNW samples, the sheet resistance was measured up to 10^4^ cycles. For GCA_1:1_, sheet resistance was increased by mere 0.2% during 10^4^ cycles. Increases in sheet resistances of 5%, 10%, and 55% were measured for GCA_1.25:1_, GCA_1.5:1_, and GCA_2:1_, respectively. These results show that GCA helps improve the mechanical properties of AgNW electrodes. It can be confirmed that the AgNP size determined according to the ratio of cysteamine and AgNP has respective advantages in the change in sheet resistance over time and the durability of the bending test.

**Figure 7:**
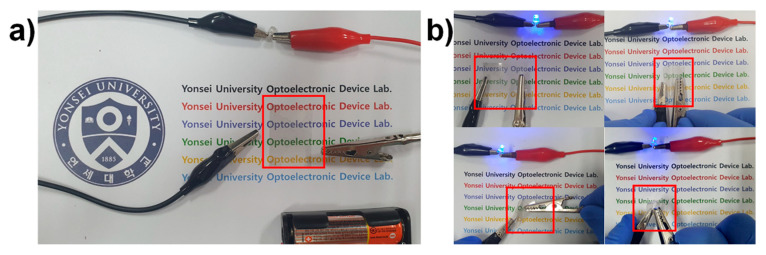
Demonstration of the mechanical robustness of the GCA–AgNW film. (**a**) Off state. (**b**) On state bending/deforming test of GCA–AgNW.

We performed the light emitting diode (LED) ON/OFF and folding tests with a serial circuit with transparent and flexible GCA–AgNW as a switch. Figure 7a shows the printed background letters in the OFF state, and Figure 7 shows that the LED circuit works well even when the film is arbitrarily bent and deformed. It is expected that GCA–AgNW can be applied as transparent electrodes with excellent flexibility.

The charged and flexible GO sheets in solution could adhere and wrap around the AgNWs and solder the inter-AgNW junctions, reducing sheet resistance and surface roughness, preventing oxidation, and greatly improving mechanical properties [28,29]. The GCA was synthesized by evenly distributed AgNPs on the GO sheet by chemical bonding using cysteamine. Thus, the GCA-AgNW network can be formed on PET with high optoelectronic performance and high mechanical robustness. Furthermore, the strong bonding and outstanding toughness of GCA could knot-tie the AgNW networks to prevent sliding inter-AgNW and thus allow high flexibility.

**Figure 8:**
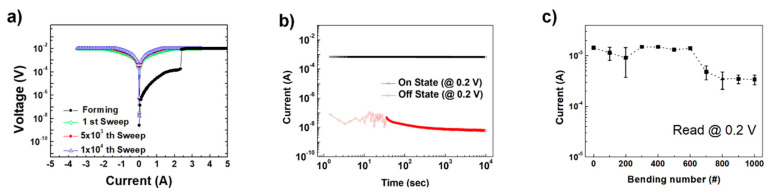
Memory characteristics of the Pt/GO/PEDOT:PSS/GCA_1:1_–AgNW/PET structure ReRAM. **(a**) I–V curve, (**b**) retention performance data, and (**c**) bending test results.

Figure 8a shows the measured I–V curves of the Pt/GO/PEDOT:PSS/GCA_1:1_–AgNW/PET structure using the GCA–AgNW layer as an electrode. It was confirmed that the device exhibited stable WORM characteristics during 10^4^ sweeping operations, and the average ON/OFF ratio was ~1.48 × 10^3^. Figure 8b shows the device durability test when the read voltage of 0.2 V is applied to the sample with ON and OFF states. In the ON state, it is observed that the current of approximately 6.9 × 10^−4^ A stably flows for 10^4^ s. In the OFF state, initially, the current flow is not stable; however, after 10 s, the current of approximately 10^−8^ A flows stably. These results confirm that the memory manufactured by GCA_1:1_ electrode can operate very stably. Figure 8c shows that the ON state current stays stable up to 600 bending cycles.

## 4. Conclusions

In this study, GCA–AgNW transparent and flexible electrodes were fabricated by depositing GCA on AgNW electrodes, and optical, electrical, and mechanical properties were analyzed. The GCA–AgNW electrode fabricated on the AgNW electrode shows excellent optical transmittance of 80% or more in the visible region (550 nm) and shows excellent properties as a transparent electrode with the FOM value of up to 116.13 in the 20–40 Ω/◻ region. The synthesis of GCA was demonstrated using EDS, FT-IR, and Raman. The structure of GCA–AgNW before and after heat treatment was observed using TEM, and the change according to the cysteamine ratio was observed. The structure of GCA–AgNW before and after heat treatment was checked using AFM and SEM to determine the cause of performance improvement. It was confirmed that the detrimental oxidation of AgNWs over time was considerably repressed, and the mechanical robustness was highly improved. In order to confirm the possibility of application of the manufactured GCA-AgNW electrode as a flexible device, a Pt/GO/PEDOT:PSS/GCA-AgNW structure ReRAM was fabricated and electrical characteristics were evaluated. The completed ReRAM ON/OFF ratio is ~1.48 × 10^3^, which confirms that GCA–AgNW has a potential to be used as a transparent and flexible electrode.

## Supplementary Materials

The following are available online at https://www.mdpi.com/2079-4991/10/12/2352/s1. Figure S1: Raman spectra of GO and GCA. Figure S2: FT-IR data of GO and GCA.
