# Supplementary material for: Flexible Transparent Electrode Characteristics of Graphene Oxide/Cysteamine/AgNP/AgNW Structure
Source: Nanomaterials (Basel). 2020 Nov 27;10(12):2352. doi: 10.3390/nano10122352 (PMC7760301; doi:10.3390/nano10122352)
Supplement: Supplementary file 1 [file nanomaterials-10-02352-s001.pdf]

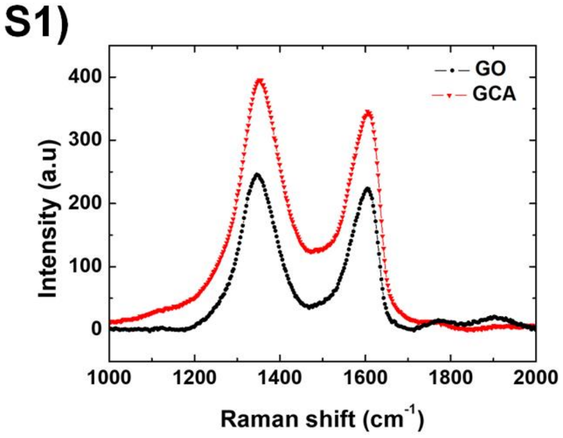

Fig S1. Raman spectra of Graphene oxide (GO) and GO–cysteamine–Ag nanoparticles (GCA).

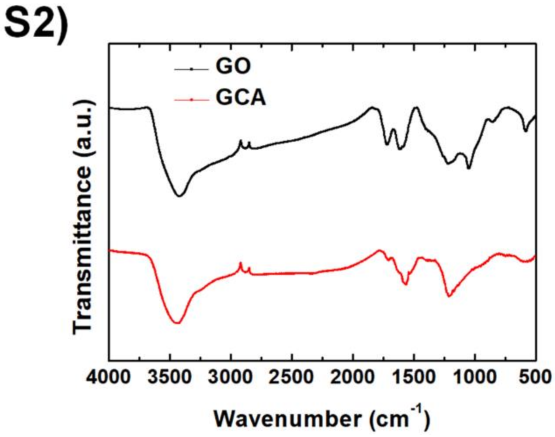

Fig S2. Fourier-transform infrared spectroscopy data of GO and GCA.
